# Quality assurance in cytology reporting in Ghana: an urgent call

**DOI:** 10.3332/ecancer.2023.1495

**Published:** 2023-01-09

**Authors:** Kofi Effah, Joseph Emmanuel Amuah, Ethel Tekpor, Comfort Mawusi Wormenor, Bernard Hayford Atuguba, Sodzi Sodzi-Tettey, Stephen Danyo, Patrick Kafui Akakpo

**Affiliations:** 1 Catholic Hospital, Battor, P. O. Box 2, Battor, via Sogakope, Volta Region, Ghana; 2 School of Epidemiology and Public Health, Faculty of Medicine, University of Ottawa, Ottawa, Ontario K1H 8M5, Canada; 3 Institute for Healthcare Improvement, State Street, 18th Floor, Boston, MA 02109, USA; 4 Department of Pathology, School of Medical Sciences, University of Cape Coast, Cape Coast Teaching Hospital, Cape Coast, P. O. Box CT 1362 CPAE COAST Ghana

**Keywords:** reporting, quality, cytology

## Abstract

**Introduction:**

In Ghana, the Papanicolaou (PAP) smear remains central to cervical cancer screening although human papilloma virus testing is recommended. The success of the PAP smear however depends on stringent quality processes. Unfortunately, PAP smear reporting in Ghana is uncoordinated with no clear quality guidelines.

**Methods:**

We applied quality guidelines to all PAP smear diagnoses of high-grade squamous intraepithelial lesion (HSIL) at Catholic Hospital Battor from 1 June 2016 to 31 August 2021. Available slides were independently reviewed by two pathologists, colposcopy findings were correlated with PAP smear results and histology cytology correlation was carried out after loop electrosurgical excision procedure (LEEP).

**Results:**

Of 17 women with HSIL, 3 available slides were reviewed and found to be normal (negative for intraepithelial lesion or malignancy), obviating the need for LEEP. Of the 11 that had LEEP after colposcopy, cytology histology correlation revealed that 54.6% (6) had no dysplasia, 27.3% (3) were cervical intraepithelial neoplasia (CIN) II and 18.2% (2) were CIN III. Cytology, colposcopy correlation showed that (out) of the 17 women, 52.9% (9) had no lesions, 29.4% (5) had minor changes and 17.7% (3) had major changes on their cervix. Of the nine that had no lesions on colposcopy, five had LEEP. Of these five, dysplasia (at least CIN II) was revealed in three (60%).

**Conclusion:**

The lack of quality processes in PAP smear reporting results in a high false positive rate with overtreatment of patients. Quality measures need to be adopted for the reporting of PAP smears in Ghana if gains are to be made in the fight against cervical cancer.

## Introduction

Cervical cancer is the second leading cause of cancer deaths in women in Ghana [1]. The Catalan Institute of Oncology (ICO)/International Agency for Research on Cancer (IARC) Information Centre on HPV and Cancer estimates that 2,797 women are diagnosed with cervical cancer and 1,699 die from the disease annually in Ghana [1]. They rank cervical cancer as the number two (2) cancer among women in Ghana and again as the second most frequent cancer among women between 15 and 44 years of age [1]. High-risk human papilloma virus (HPV) infection is established as the cause of cervical cancer and it is known that invasive cancer arises over a prolonged number of years with the peak in risk period reported to be at about 35–55 years of age [2]. Because the progression of high-risk HPV infection to cervical cancer occurs over a prolonged duration, disease progression is characterised by morphological changes within the cervical cells and at the molecular level that allow for cervical precancer screening [2].

Access to effective screening and preventive treatment in high-income countries has resulted in differences in incidence rates of cervical cancer between high- and low-income countries where there is a lack of these services [3]. Until recently, the Papanicolaou (PAP) smear was the ideal screening test in high-income countries. It allowed for the efficient and inexpensive screening of all women regardless of their social situation [3]. It is considered the most successful cancer screening test, resulting in a drastic reduction in the incidence of cervical cancer in all countries that run effective screening programmes [4]. In Ghana, resource limitations mean that clients pay out of pocket to be screened and thus decide what type of cervical cancer screening service to have. Thus, the PAP smear remains pivotal in cervical cancer screening. Its proven ability to reduce cervical cancer incidence means that it remains a widely used cervical cancer screening test in Ghana. Apart from being used as a standalone primary cervical cancer screening tool, it is also used as an aid for triaging patients who test positive with other screening modalities such as visual inspection with acetic acid and high-risk HPV testing. In these settings, it is used as a triaging tool for follow-up of patients who test ‘positive’ for other modalities.

To attain the results achieved by high-income countries using PAP smear screening requires, among other things, robust quality control (QC) and quality assurance (QA) in cytology. ‘The set of measures designed to ensure the accuracy of interpretation and reporting of cervical smears is termed *Quality Control (QC)*.’ ‘The process of building quality control into a system is termed *Quality Assurance (QA)’.* Continuous quality improvement (CQI) refers to all procedures aimed at monitoring, correcting and improving the processes and outcomes of health services. It is designed to maintain and improve quality of cytological diagnostic services [6]. The immense benefit derived from the use of the PAP smear for cervical cancer screening was due to in-built CQI processes adopted by countries that used the PAP smear for primary screening. In the United States of America, QA/QC in cytology was further reinforced by the amendment of the Clinical Laboratory Improvement Act as far back as 1988 [7]. The Act details the rules, procedures and processes that must be carried out by laboratories to be certified to practise cytology screening [8]. Elsewhere in the European Union, a team developed a document to guide the practice of cytology in relation to the PAP smear [5].

CQI minimises reporting errors and thus ensures accurate reporting so that patients are not overtreated or undertreated [5]. To assure quality, there must be continuous measurement and monitoring of laboratory performance against an agreed standard. Accuracy is the most critical measure of quality in cytology reporting. Accuracy ensures that false negative and false positive results are eliminated in cytology reporting. An accurate cytological diagnosis is one that agrees with or is same as the diagnosis on the Gold standard (tissue in cytology). This means that cytology histopathology correlation is an objective way of assessing the quality of cytological diagnosis. Other accepted standards that have been used include colposcopic findings and consensus diagnosis on the same cytological slide [5–7]. To ensure that there is accurate reporting of smears, there is hierarchical reporting of cytology slides with slides screened first by cytotechnicians/scientists or by automated screeners. After this, a proportion of normal slides is selected at random, and all borderline and abnormal slides are selected for review by a cytopathologist or by a person designated to do so by the pathology laboratory [5–8]. In addition to the methods described, others such as rapid review of all slides reported as negative, supervisory review of all cases with selected clinical presentations, review of all previous smears of women who have a positive report, seeding of routine cytology workload with known positive slides to pick up poor reporting, statistical monitoring of laboratory performance and cytology/histology comparison are mandatory in most countries with functional PAP based screening programmes.

With no national screening programme, Ghana has no such quality measures in place and slides are reported countrywide by scientists and pathologists without QC, QA and CQI. The effect of this on quality of reporting has not been assessed. Our aim was to bring attention to the non-existence of QC, QA and CQI and its resultant dire consequences for patients in PAP smear cytology in Ghana. We applied selected applicable quality measures including slide review by independent pathologists, correlation of colposcopic findings with PAP smear cytology diagnosis and correlation of histopathological diagnosis after loop electrosurgical excision procedure (LEEP) with PAP smear cytology diagnosis, to all cases with high-grade squamous intraepithelial lesion (HSIL) diagnosis on PAP smear cytology. To assure quality and avoid unnecessary treatment, the process of QC, QA and CQI was initiated by the Cervical Cancer Prevention and Training Center (CCPTC) unlike usual QC, QA and CQI processes that are initiated by the reporting laboratories.

## Methods

Catholic Hospital Battor (CHB) serves as the District Hospital for North Tongu District, one of the 18 administrative districts/municipalities in the Volta Region of Ghana. The hospital serves a rural population but also receives many clients from urban settings. CCPTC was established in May 2017 and is involved in cervical precancer screening, follow-up, treatment of precancerous lesions of the cervix as well as training of health workers in cervical cancer prevention skills. Before May 2017, cervical precancer screening was performed in the Gynaecology Department of the hospital.

In this descriptive cross-sectional study, we reviewed all 17 clients who were diagnosed as HSIL through cytology and seen at the Gynaecology Department, CHB, and at the Cervical Cancer Prevention and Training Centre in CHB from 1 June 2016 to 31 August 2021. Colposcopy using Colposcopy with the mobile colposcope, Enhanced Visual Assessment (EVA) system (MobileODT, Tel Aviv, Israel), was carried out on all the 17 clients. Colposcopy reports of all patients with positive cytology results were retrieved and analysed. This process followed international guidelines for QC and QA in PAP cytology reporting. These processes included cytology slide second opinion, cytology colposcopy correlation and cytology histology correlation which is the gold standard. This was guided by an algorithm designed to assure quality at the CCPTC. All slides except one were reported according to the BETHESDA system [8, 9].

Of the 17 clients that formed the basis for this study, 11 were treated by LEEP and their histopathology reports were reviewed. This informed the decision to conduct independent cytology reviews for future HSIL cytology reports prior to definitive treatment. Of the remaining six patients, reports of three positive cytology slides were retrieved and the reporting cytologist or pathologist contacted on phone about the need for a review as part of the QA process. QA in cytology was carried out through a review of these three positive slides by two other pathologists independently. Slides for the other three clients could not be reviewed by the end of the data collection period because for two of the slides, despite repeated calls to the reporting laboratories, they did not bring their slides for independent review and for the one remaining slide, the responsible cytologist was unable to provide the slide for independent review.

Cytology colposcopy correlation was carried out by comparing the cytological findings with findings at examination during colposcopy. When a lesion was found and a biopsy done, cytology histology correlation was carried out after histopathological assessment of the tissue taken at colposcopy. Results were entered into an excel spreadsheet and analysed.

Specifically, we determined the following in the statistical analyses:

the proportion of eligible clients with an original HSIL cytology diagnosis whose results remained HSIL following a secondary review by two independent pathologists.

the proportion of clients with an original HSIL cytology diagnosis whose colposcopy findings were positive for high-grade cervical lesions (major change).

the proportion of eligible clients with an original HSIL cytology diagnosis whose histopathology findings were positive for cervical intraepithelial neoplasia (CIN) II and above.

the proportion of eligible clients with a positive colposcopy finding (presence of lesions on the cervix) whose histopathology findings were positive (diagnosis of at least CIN II).

the proportion of eligible clients with no lesion seen at colposcopy whose histopathology findings were positive (diagnosis of at least CIN II).

Other demographical parameters and salient details such as high-risk HPV status and HIV status were also analysed for the 17 patients.

Continuous variables such as age are summarised using mean and standard deviation as they were symmetric. Categorical variables like level of education, HIV status, marital status, etc., are analysed using counts and proportions. No statistical hypothesis tests were conducted. All statistical analyses were conducted in STATA version 14.

## Results

A total of 17 women (Table 1) with PAP diagnosis of HSIL were seen at the CCPTC of the CHB following cytology diagnosis out of which 3 were able to provide their slides to confirm HSIL diagnosis.

All seventeen (17) of them had colposcopy. Three (3) patients had their cytological diagnosis changed to normal following independent review. This was because only three (3) cytology slides were available to be reviewed and thus only the three (3) had an independent review of slides. Two (2) were lost to follow-up. Eleven (11) of them had LEEP.

### Sociodemographic characteristics

The mean age of the 17 women was 50.5 ± 12.9 years, 58.8% (10) had used contraception in the past, none of them was using contraception at the time of clinical review and the majority (53%) had fewer than three children. The majority (53%) were married or had a steady partner, while the rest were divorced (23.5%), widowed (17.6%) or single (5.9%). The majority of women completed Junior High School or higher (64.7%) and almost all of them (94.1%) were Christians.

### Clinical characteristics

A majority (70.6%) of the women were HIV negative and the rest had an unknown HIV status. At colposcopy, 52.9% (9) had no lesions, 29.4% (5) had minor changes on their cervix and 17.7% (3) had major changes on their cervix. The transformation zone (TZ) types from colposcopy also showed that 88.2% were Type 3, 5.9% were Type 2 and 5.9% were Type 1. Majority 87.5% (7) of the 8 clients who had a positive colposcopy finding had a TZ type of T3 (the entire circumference of the squamocolumnar junction was not visible; partly or fully endocervical) [10].

**Figure 1.**
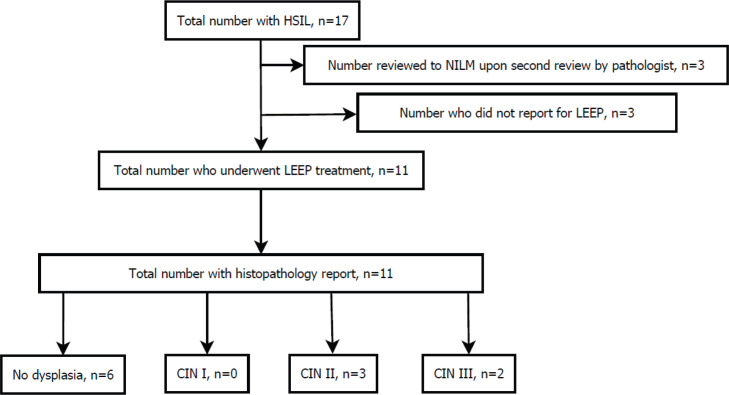
A flowchart depicting the flow of 17 women from initial HSIL diagnosis, secondary review by independent pathologists, treatment and histopathology.

Of the 17 women (Figure 1), 3 of them did not come for review in spite of repeated calls and so were deemed lost to follow-up. Of the remaining 14, 3 patients who were yet to have colposcopy done had their slides reviewed by independent pathologists and their initial diagnosis changed to negative for intraepithelial lesion or malignancy (NILM) and thus the corresponding clients did not require treatment. The other 11 patients underwent LEEP, and their samples were taken for histopathology. This was prior to the CCPTC deciding to ask for second opinion on all HSIL slides. Of these 11, 54.6% (6) had no dysplasia, 27.3% (3) were CIN II and 18.2% (2) were CIN III.

### Concordance between cytology, colposcopy and histology

Of the 11 women who were treated with LEEP, 54.6% (6) had a lesion seen at colposcopy. Of these 6, 33.3% (2) had a histopathology result of at least CIN II. Of the rest (5) that had no lesion at colposcopy, 40% (2) had no dysplasia and 60% (3) were determined to be CIN II. All these three clients had a TZ type of T3.

Out of 3 clients classified as NILM upon secondary review by independent pathologists, 33.3% (1) of them had a lesion at colposcopy (minor change) and all three (3) had TZ type of T3.

## Discussion

**Figure 2.**
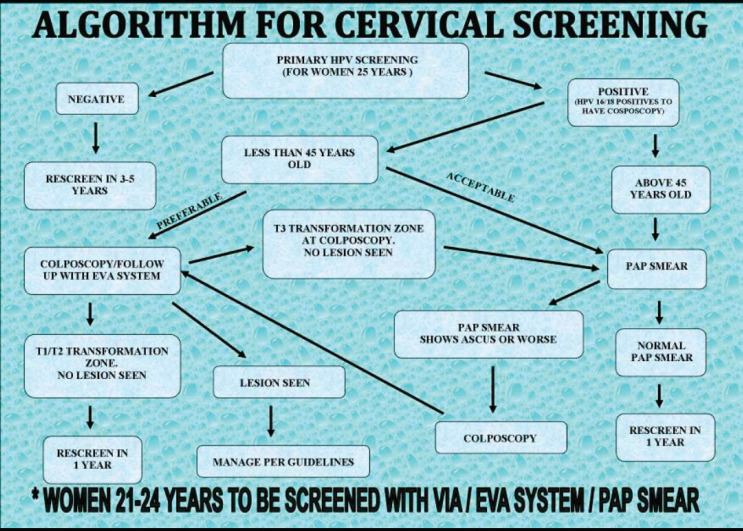
Cervical precancer screening algorithm at the CCPTC, Battor.

In cervical precancer screening, Battor moved from primary screening with cytology to HPV DNA testing in June 2016. Many institutions in Ghana still use primary screening with cytology. However, many women still prefer primary screening with cytology even after all other available options have been explained to them. At the CCPTC, our algorithm (Figure 2) demands follow-up with cytology when a woman is high-risk HPV positive, has TZ type 3 (squamocolumnar junction not [fully] visible, partly or fully in the endocervical canal) but no lesion or a low-grade lesion is seen on colposcopy [10]. This is to help rule out a high-grade lesion in the endocervical canal which is not visible at colposcopy because of the TZ type. As a leading comprehensive precancer and cancer care centre, the CCPTC still receives women referrals for follow-up (colposcopy) after primary screening with cytology in peripheral/other facilities. It is therefore essential that quality is assured to avoid overtreatment and undertreatment of patients.

The BETHESDA system for cytology reporting is accepted as a standardised and reproducible method of reporting cytology smears. This method is constantly improved to include salient parameters that are important for cervical precancer screening and treatment. The parameters to report are clearly spelt out in this system and it allows the comparison of reports for quality purposes [10]. In Ghana, it is expected that all PAP smears are reported according to the Bethesda system though not enforced. In this study, all reports except one were according to the Bethesda system.

Though guidelines for PAP reporting stipulate that there should be a system for reviewing 10% of all negatives slides and also for reviewing all positive smears [5–8], none of the centres reporting PAP smears reported the existence of such a system and in all cases the reports were signed out by a single individual. For three (3) cases with an initial diagnosis of HSIL that were yet to have LEEP where slides were reviewed by two other pathologists in accordance with QA and QC measures which stipulate second opinion in all HSIL diagnosis, the diagnosis was subsequently changed to normal (negative for intraepithelial lesion of malignancy) after the independent review by two pathologists with 100% agreement in all three cases. This obviated the need for LEEP. This high false positive rate is worrying especially because it would have resulted in unnecessary treatment. The alternative (false negative) can have more dire consequences especially in our setting where many patients only get screened once in a lifetime. This was however not explored in our study.

**Figure 3.**
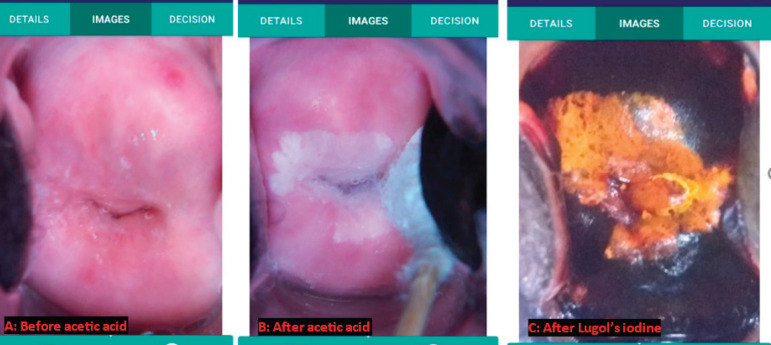
Case number 3, showing good correlation between cytology, colposcopy and histopathology. CareHPV – positive (for high-risk HPV); Pap – HSIL; Colposcopy – major change (dense acetowhitening); Histopathology – CIN III.

HSIL is typically managed by LEEP. This is a procedure that uses a wire loop and electric current to excise part of the cervix [11]. The LEEP is usually preceded by colposcopy to direct the extent of the excision. At colposcopy, major changes (like dense acetowhitening, coarse mosaicism, coarse punctuations) are expected which can be biopsied to confirm a diagnosis of CIN II or CIN III before the LEEP (Figure 3). In low-resource settings where the lack of funds often influences management, LEEPs may be performed without biopsies of cervical lesions. Patients are often lost to follow-up and thus a see and treat approach is justifiable in our setting. When no lesions are found on the ectocervix with a cytology report of HSIL, and there is TZ type 3 (the squamocolumnar junction is in the endocervical canal and not [fully] visible), the assumption is that the high-grade lesion is in the endocervical canal and a LEEP is still recommended. Almost all cervical cancers develop in the TZ so colposcopy is reassuring when the full TZ is visible, TZ type 1 or 2 (Type 1; the entire circumference of squamocolumnar junction is visible; fully ectocervical or Type 2; the entire circumference of squamocolumnar junction is visible; partly or fully endocervical). The common complications of LEEP are cervical/vaginal bleeding, infection, cervical stenosis and cervical incompetence leading to miscarriages and preterm labour [12, 13]. Unusual complications of LEEP are postoperative peritonitis, vesicovaginal fistula and lower urinary tract injury [14–17]. The risk of serious complications from LEEP means the indication must be correct. A diagnosis of HSIL should therefore not be taken lightly.

In the 2011 International Federation of Cervical Pathology and Colposcopy (IFCPC) colposcopic terminology of the cervix, minor changes (Grade 1) refer to thin acetowhite epithelium, irregular, geographic border, fine mosaic and fine punctuation. Major changes (Grade 2) refer to dense acetowhite epithelium, rapid appearance of acetowhitening, cuffed gland openings, coarse mosaic, coarse punctuation, sharp border, inner border sign and Ridge sign (Table 2) [10, 18]. When cytology was compared with colposcopy findings, it was revealed that out of 17 women, who had colposcopy 51.9% (9) had no lesions, 29.4% (5) had minor changes on their cervix and 17.7% (3) had major changes on their cervix [10, 18]. Of the 51.9% (9) that had no lesions on colposcopy, only 5 had LEEP which revealed dysplasia (at least CIN II) in 3 (60.0%) of the 5. This confirms that colposcopy may not reveal some lesions detected by PAP, reinforcing the current TZ (especially for type 3) adequacy categorisation system used at the CCPTC. Of the 5 women who had CIN2/CIN3 on histopathology after LEEP, 1 (20.3%) had major changes at colposcopy which correlates well with cytology and histopathology reports (Figure 3). One (20.0%) had minor changes at colposcopy. Though this did not correlate well with the cytologic or histologic diagnosis, this is understandable because with a TZ type 3, a higher-grade lesion could have been in the endocervical canal. This also applies to all the 3 (60.0%) cases in which no lesions were seen at colposcopy, as they all had TZ type 3.

From June 2016 to August 2021, there were 17 reports of HSIL. Eleven of them had LEEPs. The histopathology report of six of them came as ‘no dysplasia’. Of these none of the cytology reports of HSIL came from cytotechnologists, while three came from a Biomedical Scientist and pathologists, respectively. In November 2020, a decision was taken to get two independent cytopathologists to review the slides whenever a diagnosis of HSIL was made and no lesion was seen at colposcopy. This was to avoid LEEPs that were not indicated. All three of such HSIL slides reviewed were changed to NILM and LEEPs abandoned. Of these, initial cytology report of HSIL came from a cytotechnologist on one occasion, a Biomedical Scientist on one occasion and a pathologist on one occasion.

In this review, out of 11 patients who had the gold standard for QA in PAP reporting (cytology-histology correlation) 54.6% did not show any lesion on histology suggesting a high and unacceptable error rate in the reporting of PAP smears. The gold standard for QA in cytology is cytology-histology correlation, but histology requires a biopsy/LEEP which may be overtreatment and come with severe complications and unnecessary costs.

It suggests that QC, QA and CQI procedures in relation to PAP reporting must be stringently enforced to prevent overtreatment of patients. This should be augmented with easier methods such as cytology colposcopy correlation keeping in mind the limitations of this method. In our study, the mean age of the 17 women was 50.5 ± 12.9 years. It is reported that the squamocolumnar junction recedes into the endocervical canal and is not visible for majority of women after 45 years (TZ type 3). This is in line with the high proportion of transformation type 3 seen in our study [19]. Again cytology histology correlation must be adopted by all reporting laboratories to ensure and guarantee CQI. This should be tied in with proper training of both pathologists and cytotechnicians as stipulated in quality guidelines [6]. There is currently no certified subspeciality training in cytopathology or cytotechnology in Ghana. The certification of reporting laboratories should also be prioritised to ensure that those reporting PAP smears are adequately trained.

Without an in-house pathology service, the CCPTC relies on external laboratories for cytology and histology services. This is coordinated by the CCPTC through courier and electronic reports. These services include working visits to the CCPTC. This means that the CCPTC relies on QC and CQI programmes instituted at the various laboratories that provided services for the CCPTC. Many laboratories in Ghana however lack comprehensive CQI programmes. This is compounded by the lack of communication between the end user (CCPTC) and the pathology laboratory. To ensure that all the methods available for cytology CQI are employed for cytopathology reporting, the CCPTC maintains an open communication channel to all its pathology service providers to ensure that all positive cases are correlated with the colposcopic and histopathological findings where available, in accordance with international guidelines for CQI [5–7]. Following the initiative by CCPTC, our primary reporting pathology laboratory instituted QA, QC and CQI programme and continues to receive feedback on histological diagnosis for CQI.

In the absence of molecular tests with high positive predictive values in detecting premalignant lesions of the cervix (CIN 2+), cytology will remain part of cervical cancer screening programmes especially for follow-up of high-risk HPV positives. Also because of the high positivity rate of high-risk HPV using HPV DNA testing for women less than 25 years when most of these lesions are transient, primary screening with cytology will remain useful due to its high specificity compared to HPV DNA testing. This means it is important to make cytology accurate using QA and QC methods such as intradepartmental reviews and finally cytology, colposcopy and histology correlation. This will ensure CQI of the reporting pathologists. This is crucial to reduce the number of false positives that may be aggressively treated wrongly.

## Conclusion

The inclusion of QC and QA processes in the routine practice of cytology is crucial for the success of cervical cancer prevention in Ghana. It is important that routine hierarchical reporting and review of a proportion of all negative cytology slides is carried out. The process should involve review of all positive slides by experienced cytopathologists. This should be carried out to prevent overtreatment of false positives and also reduce the scenario where high-grade lesions are missed. Again cytology-colposcopy-histology correlations should be used carefully if used routinely as part of QC in cervical cancer screening. To improve quality, there should be regular multidisciplinary team meetings where the screening teams (nurses, gynaecologists) meet with cytotechnologists and pathologists to review cases. This will assure quality and guarantee its improvement.

## Conflict of interest

The authors declare no conflict of interest.

## Funding

No funding was received for this project.

## Ethics & consent

Ethical approval was granted by the Ethical Review Committee of CHB.

## Author contributions

Conceptualisation: KE, PKA, ET, CMW, BHA

Screening and data collection: ET, KE, CMW, BHA

Data management and formal analysis: JEA, ET, SD, KE, CMW, BHA

Writing – original draft: PKA, JEA, KE, SST, ET

All the authors read and approved the manuscript in its current form.

## List of abbreviations

PAP smear, Papanicolaou smear; NILM, Negative for intraepithelial lesion or malignancy; HSIL, High-grade squamous intraepithelial lesion; CIN, Cervical intraepithelial lesion; LEEP, Loop electrosurgical excision procedure; EVA, Enhanced visual assessment; QA, Quality assurance; QC, Quality control; CQI, Continuous quality improvement; TZ, Transformation zone; CHB, Catholic Hospital Battor; CCPTC, Cervical Cancer Prevention and Training Center; HPV, Human papilloma virus.

## Figures and Tables

**Table 1.** Sociodemographic and clinical characteristics of clients diagnosed as HSIL (*n* = 17).

Characteristic	Estimate
**Age, mean (standard deviation)**	50.5 (12.9)
**Marital status, % (*n*)** ** Single** ** Has a steady partner** ** Married** ** Divorced** ** Widowed**	5.9 (1) 11.8 (2) 41.2 (7) 23.5 (4) 17.6 (3)
**Current contraceptive use, % (*n*)**	0.0 (0)
**Past contraceptive use, % (*n*)**	58.8% (10)
**Number of children** ** 0** ** 1** ** 2** ** 3+**	11.8 (2) 29.4 (5) 11.8 (2) 47.0 (8)
**Highest level of education, % (*n*)** ** No formal education** ** Elementary education** ** Secondary education** ** Tertiary education**	17.7 (3) 17.7 (3) 29.4 (5) 35.3 (6)
**Religious faith, % (*n*)** ** Christian** ** Muslim**	94.1 (16) 5.9 (1)
**HIV status, % (*n*)** ** Negative** ** Unknown**	(12) 29.4 (5)
**Previous cervical screening, % (*n*)**	100.0 (17)
**Adequate view with mobile colposcopy, % (*n*)**	100.0 (17)
**TZ types, % (*n*)** ** TZ 1** ** TZ 2** ** TZ 3**	5.9 (1) 5.9 (1) 82.4 (15)
**Colposcopy findings (EVA mobile colposcopy), % (*n*)** ** No lesions** ** Minor changes** ** Major changes**	52.9 (9) 29.4 (5) 17.6 (3)

**Table 2.** *2011 IFCPC Nomenclature* [18].

2011 IFCPC colposcopic terminology of the cervix [18]			
**General assessment**		Adequate/inadequate for the reason … (i.e.: cervix obscured by inflammation, bleeding, scar) Squamo-columnar Junction visibility: completely visible, partially visible, not visible Transformation zone types 1,2,3	
**Normal colposcopic findings**		Original squamous epithelium: Mature Atrophic Columnar epithelium Ectopy Metaplastic squamous epithelium Nabothian cysts Crypt (gland) openings Deciduosis in pregnancy	
**Abnormal colposcopic findings**	**General principles**	**Location of the lesion**: Inside or outside the T-zone, Location of the lesion by clock position **Size of the lesion**: Number of cervical quadrants the lesion covers, Size of the lesion in percentage of cervix,	
	**Grade 1 (Minor)**	Thin aceto-white epithelium Irregular, geographic border	Fine mosaic, Fine punctation
	**Grade 2 (Major)**	Dense aceto-white epithelium, Rapid appearance of acetowhitening, Cuffed crypt (gland) openings	Coarse mosaic, Coarse punctuation, Sharp border, Inner border sign, Ridge sign
	**Non specific**	Leukoplakia (keratosis, hyperkeratosis), Erosion Lugol’s staining (Schiller’s test): stained/non-stained	
**Suspicious for invasion**		Atypical vessels **Additional signs**: Fragile vessels, Irregular surface, Exophytic lesion, Necrosis, Ulceration (necrotic), tumor/gross neoplasm	
**Miscellaneous finding**		Congenital transformation zone, Condyloma, Polyp (Ectocervical/ endocervical) Inflammation,	Stenosis, Congenital anomaly, Post treatment consequence, Endometriosis

**Table 3.** Details of colposcopy and histopathology report of 17 clients diagnosed as HSIL.

Client number	Colposcopy findings	TZ type	Histopathology report
**1**	Minor changes	T1	No CIN
**2**	No lesions	T3	CIN II
**3**	Major changes	T3	CIN III
**4**	Minor changes	T3	No CIN
**5**	No lesions	T2	Lost to follow-up
**6**	Minor changes	T3	Pap revised to NILM
**7**	Minor changes	T3	CIN III
**8**	No lesions	T3	CIN II
**9**	No lesions	T3	Cytologist could not provide slide for review. Negative for hrHPV.
**10**	Major changes	T3	Lost to follow-up
**11**	Minor changes	T3	No CIN
**12**	Minor changes	T3	No CIN
**13**	No lesions	T3	Pap revised to NILM
**14**	No lesions	T3	CIN II
**15**	No lesions	T3	No CIN
**16**	Major changes	T3	No CIN
**17**	No lesions	T3	Pap revised to NILM

## References

[1] Ghana; Human Papillomavirus and Related Cancers, Fact Sheet 20212021-10-22 Document.

[2] Schiffman M, Castle PE, Jeronimo J (2007). Human papillomavirus and cervical cancer. Lancet.

[3] Tsu V, Jerónimo J (2016). Saving the world’s women from cervical cancer. N Engl J Med.

[4] Shaw PA (2000). The history of cervical screening I: the Pap test. J Soc Obstet Gynaecol Can.

[5] Branca M, Coleman DV, Marsan C (2021). Quality Assurance and Continuous Quality Improvement in Laboratories Which Undertake Cervical Cytology.

[6] Branca M, Longatto-Filho A (2015). Recommendations on quality control and quality assurance in cervical cytology. Acta Cytol.

[7] Heher YK, Chen Y, VanderLaan PA (2017). Measuring and assuring quality performance in cytology: a toolkit. Cancer Cytopathol.

[8] https://www.ecfr.gov/cgi-bin/text-idx?SID=1248e3189da5e5f936e55315402bc38b&node=pt42.5.493&rgn=div5#_top.

[9] The Bethasda System for Cervical Cytology Reporting.

[10] Quaas J, Reich O, Küppers V (2014). Explanation and use of the rio colposcopy nomenclature of the IFCPC (International federation for cervical pathology and colposcopy): comments on the general colposcopic assessment of the uterine cervix: adequate/inadequate; squamocolumnar junction; transformation zone. Geburtshilfe Frauenheilkd.

[11] Guido R, Lonky NM, Diedrich J (2014). Secondary prevention of cervical cancer part 3: evidence-based management of women with cervical intraepithelial neoplasia. Clin Obstet Gynecol.

[12] Garcia F, Hatch KD, Berek JS, Berek JS, Novak E (2012). Intraepithelial disease of the cervix, vagina, and vulva. Berek & Novak’s Gynecology.

[13] Massad LS, Jones HW, Rock JA (2015). Cervical cancer precursors and their management. Te Linde’s Operative Gynecology.

[14] Varras M, Akrivis C, Anastasiadis A (2012). Peritonitis due to iatrogenic colpotomy after large loop excision of the transformation zone (LLETZ) in a patient with cervical intraepithelial neoplasia III: our experience of a rare case with review of the literature. Eur J Gynaecol Oncol.

[15] Cho SS, Kang WD, Kim SM (2011). A case of vesicovaginal fistula after loop electrosurgical excisional procedure. Korean J Obstet Gynecol.

[16] Ghassani A, Andre B, Simon-Toulza C (2014). Vaginal evisceration: an unexpected complication of conization. Case Rep Obstet Gynecol.

[17] Contag SA, Wilson TO (2004). Ureteral injury during cold-knife cervical conization. J Pelvic Med Surg.

[18] Bornstein J, Bentley J, Bösze P (2012). Colposcopic terminology of the International Federation for cervical pathology and colposcopy. Obstet Gynecol.

[19] Acheampong LK, Effah K, Amuah JE (2021). Determining the prevalence of high-risk human papillomavirus infection using a novel cervical precancer screening approach in incarcerated women at the Nsawam Medium Security Prison, Ghana ecancer. Ecancer Med Sci.

